# Effectiveness of Spinal Cord Stimulation in the Treatment of Lumbar Spine Pain Syndromes

**DOI:** 10.3390/medicina62050816

**Published:** 2026-04-24

**Authors:** Sebastian Podlewski, Rafał Morga, Jacek Antecki, Piotr Dubiński, Natalia Gołębiowska

**Affiliations:** 1 Department of Neurosurgery and Spine Surgery, Regional Hospital in Kielce, 25-736 Kielce, Poland; 2 Department of Neurosurgery, Scanmed St. Raphael’s Hospital, 30-693 Cracow, Poland; 3 Department of Neurology, Regional Hospital in Kielce, 25-736 Kielce, Poland

**Keywords:** chronic pain, spinal cord stimulation, failed back surgery syndrome, SCS method, functional neurosurgery

## Abstract

*Background and Objectives:* Functional neurosurgery encompasses surgical interventions aimed at modulating the function of the central and peripheral nervous systems. Spinal cord stimulation (SCS), as a form of neuromodulation, is an established treatment for chronic pain and is increasingly utilized by both anesthesiologists and neurosurgeons. The aim of this study was to evaluate the effectiveness of SCS in patients with chronic neuropathic spinal pain. *Materials and Methods:* This prospective study included 42 patients who demonstrated a positive response to trial stimulation. Only patients achieving a clinically meaningful response (≥50% pain reduction) during the trial phase were included in the final analysis. Pain intensity and functional disability were assessed using the Visual Analog Scale (VAS) and the Oswestry Disability Index (ODI). All patients underwent a two-stage percutaneous implantation procedure using burst stimulation. A follow-up assessment was performed 3–6 months after implantation. *Results:* A statistically significant reduction in pain intensity was observed (*p* < 0.0001), with median VAS scores decreasing from 8 to 3, corresponding to a 62.5% reduction in pain intensity and exceeding the minimal clinically important difference (MCID) for VAS. Functional status improved significantly, with ODI scores decreasing from 74% to 38%, markedly surpassing the established MCID threshold. A clinically meaningful reduction in pain (≥50%) was achieved in the majority of patients. All patients requiring opioid analgesics at baseline discontinued their use following SCS implantation, and a reduction in overall analgesic consumption was observed across the cohort. *Conclusions:* These findings suggest that burst SCS may be an effective treatment option for carefully selected patients with chronic neuropathic spinal pain who are not candidates for conventional spine surgery. However, the results should be interpreted with caution due to the enriched study design and limited follow-up period.

## 1. Introduction

Functional neurosurgery is a subspecialty of neurosurgery focused on the modulation of central and peripheral nervous system function. Surgical techniques used in this field include deep brain stimulation, motor cortex stimulation, peripheral nerve stimulation, and spinal cord stimulation (SCS) [1,2]. In contrast to destructive procedures, such as ablative interventions targeting neural structures, electrical stimulation enables minimally invasive and reversible modulation of nervous system activity. Dorsal column stimulation, as part of functional neurosurgery, is an established treatment modality for chronic pain and is increasingly utilized by both anesthesiologists and neurosurgeons.

Chronic pain is defined as pain persisting for more than three months. It has a profound impact on multiple aspects of a patient’s life and daily functioning, often leading to significant functional and psychological impairment. Chronic pain is also frequently associated with anxiety and depression [3,4]. It may develop as a consequence of disease or injury and, due to its persistence and severity, may become not only an accompanying symptom but also the dominant feature of a patient’s clinical presentation.

With the aging of the population, pain related to degenerative disorders of the lumbar and cervical spine has become one of the leading causes of disability worldwide. Moreover, several chronic pain syndromes are consistently ranked among the top causes of disability in the general population [5,6]. In the present study, we focus on chronic spinal pain associated with advanced degenerative disc disease or postsurgical spine syndrome.

Due to the complex and multifactorial nature of chronic pain, its management often requires a multidisciplinary approach. Patient qualification for surgical intervention typically involves collaboration among neurologists, psychiatrists, anesthesiologists, and neurosurgeons. Spinal cord stimulation offers a therapeutic option for patients who are refractory to conservative treatment, develop pharmacological tolerance, or experience significant adverse effects related to analgesic medications. Furthermore, the minimally invasive percutaneous implantation of a spinal cord stimulator may be particularly beneficial for patients with significant comorbidities who are not suitable candidates for more extensive spinal surgery.

In the present study, patients qualified for SCS implantation had either failed conservative treatment or were not eligible for invasive spinal surgery due to their general medical condition.

Despite the growing body of evidence supporting the use of spinal cord stimulation in the management of chronic neuropathic pain, the effectiveness of this therapy may vary depending on patient characteristics, underlying pain etiology, and treatment protocols. Furthermore, real-world clinical data remain essential for evaluating the outcomes of SCS therapy in specific patient populations, particularly in individuals who are not suitable candidates for conventional spinal surgery or who have failed previous treatments. Therefore, further prospective clinical observations are warranted to better characterize the effectiveness of SCS in routine clinical practice.

The aim of this study was to evaluate the clinical effectiveness of burst spinal cord stimulation in a real-world cohort of patients with chronic neuropathic spinal pain who were not candidates for conventional surgical treatment, thereby addressing the limited evidence available for this specific patient population. The prospective design was chosen to enable standardized data collection and outcome assessment in a clinically relevant setting.

### Epidemiology

Low back pain is an extremely common clinical problem, and accurate differentiation between its various subtypes—such as nociplastic, neuropathic, and nociceptive pain—is essential in the diagnostic process [7]. It is estimated that more than 84% of the population will experience low back pain at least once during their lifetime, making it a significant multidisciplinary healthcare challenge.

Low back pain represents the second most common reason for primary care consultations and is the leading cause of work-related disability and sick leave among individuals under 45 years of age [8]. Although many cases resolve spontaneously, acute lumbar pain may progress to chronic pain in approximately 2–48% of patients [9].

Identification of the underlying causes of chronic pain and the development of an appropriate treatment strategy are therefore essential. A thorough understanding of spinal anatomy and pathology allows the identification of potential structural sources of pain. It is particularly important to exclude conditions that may benefit from surgical treatment, such as intervertebral disc herniation, degenerative spinal stenosis, vertebral fractures, or spinal tumors.

Patients who continue to experience persistent pain after spinal surgery may be diagnosed with postsurgical spine syndrome, currently referred to as persistent spinal pain syndrome (PSPS). This condition describes cases in which surgical treatment does not lead to satisfactory clinical improvement. Several factors may contribute to the development of PSPS, including inappropriate patient selection, suboptimal choice of surgical technique, postoperative scar formation, adhesive arachnoiditis, or loss of sagittal spinal balance. In addition, patient-related factors also play an important role. Previous studies have demonstrated that patients with depression, mood disorders, or low self-esteem tend to have poorer outcomes and may be at increased risk of developing PSPS [10].

Patients who do not respond adequately to conservative treatment, experience unsatisfactory outcomes after spinal surgery, lack clear indications for further surgical intervention, or cannot undergo conventional spine surgery due to medical contraindications may represent a group of potential candidates for treatment with spinal cord stimulation.

## 2. Materials and Methods

### 2.1. Patient Group

The prospective study included 42 patients over 50 years of age with chronic neuropathic spinal pain who were not candidates for standard surgical procedures. In 38% of cases, no clear surgical indication was identified (e.g., herniated nucleus pulposus or spinal stenosis). In 44% of patients, surgery was contraindicated due to its invasiveness or the presence of significant medical comorbidities. The remaining 18% had previously undergone unsuccessful surgical treatment, such as discectomy or spinal fusion.

The study included patients with chronic lumbar pain lasting more than 12 months who had a history of long-term use of analgesics and anti-inflammatory medications and had exhausted available conservative treatment options. Patients were diagnosed with chronic neuropathic spinal pain based on clinical presentation, imaging findings, and prior treatment history. Pain phenotype was assessed clinically, and patients were classified as having predominantly neuropathic pain based on characteristic features such as radiating pain, burning sensation, and paresthesia. Patients with predominantly nociceptive pain without a neuropathic component were not considered candidates for SCS therapy. In the preoperative period, almost all patients required strong analgesics, including tramadol or opioid medications.

All patients underwent diagnostic imaging (CT or MRI) to exclude reversible causes of symptoms that could be treated surgically and to identify potential anatomical contraindications to electrode implantation. All patients met predefined clinical criteria for chronic neuropathic spinal pain based on clinical assessment and imaging findings, thereby minimizing classification variability. Prior to qualification for surgical treatment, all patients underwent psychological evaluation to exclude hypochondriacal disorders, severe depression unrelated to chronic pain, or inadequately treated depressive disorders that could potentially influence surgical outcomes. According to current clinical recommendations, such conditions were considered contraindications to SCS therapy due to their association with poorer treatment outcomes.

Patients with a previous history of spinal cord stimulator implantation for pain management were excluded from the study.

The exact number of patients who failed trial stimulation was not recorded, which represents a limitation of the study.

All consecutive patients meeting the inclusion criteria and undergoing trial stimulation during the study period were considered for enrollment. Patients who achieved ≥50% pain reduction during trial stimulation were qualified for permanent implantation. One patient was excluded due to electrode damage, resulting in a final cohort of 41 patients.

All patients completed follow-up assessments, and no dropouts occurred during the study period. No missing data were identified in the analyzed dataset; therefore, no imputation methods or sensitivity analyses were required. All patients met predefined clinical criteria for chronic neuropathic spinal pain based on clinical assessment and imaging findings, which further minimized classification variability.

The study was conducted in the Department of Neurosurgery between January 2024 and May 2025. The study protocol was approved by the Bioethics Committee of the Świętokrzyska Medical Chamber (approval number: 52/2024). The study was based on prospectively collected clinical data and approved for retrospective analysis. All procedures were performed in accordance with the ethical standards of the World Medical Association Declaration of Helsinki.

### 2.2. Surgical Technique

All 42 patients were treated by a single surgeon. The procedure involved percutaneous implantation of a spinal cord stimulator electrode, and burst stimulation was used. Burst stimulation consists of a cyclic series of electrical impulses combining high-frequency and tonic stimulation.

Prior to surgery, patients received 10 mg of morphine administered subcutaneously approximately 30 min before the procedure, as well as antibiotic prophylaxis consisting of either 2 g of cefazolin or 600 mg of clindamycin. All procedures were performed under local anesthesia using 1% lidocaine, which allowed intraoperative programming of the stimulator to ensure appropriate coverage of the painful areas.

The stimulator electrode was inserted percutaneously using a Tuohy needle under fluoroscopic guidance with multiplanar X-ray imaging to confirm the correct electrode position. During the trial stimulation period, the electrode extension was externalized through the skin.

The treatment was performed in two stages. The first stage involved percutaneous electrode implantation followed by a trial stimulation period of 14–21 days. Only patients who achieved a clinically meaningful response, defined as ≥50% reduction in pain, were qualified for permanent implantation. This enrichment design reflects standard clinical practice; however, it limits the generalizability of the findings.

During the second stage, performed under local anesthesia, the pulse generator (stimulator battery) was implanted in the subcutaneous tissue of the right buttock.

In all patients, the procedure was performed using a spinal cord stimulation system manufactured by Abbott. Stimulation parameters were individualized for each patient based on clinical response. Burst stimulation (BurstDR) was delivered using standardized settings, including a burst frequency of 40 Hz, an intraburst frequency of 500 Hz, and a pulse width of 1000 µs. Amplitude was applied at low intensities (approximately 0.05–0.6 mA) and adjusted individually to achieve optimal clinical effect. Cycling stimulation patterns were used (e.g., 30 s on/30–180 s off) according to the manufacturer’s protocol.

### 2.3. Evaluation of Surgical Treatment Outcomes

Pain intensity was assessed before surgical treatment using the validated Polish versions of the Visual Analog Scale (VAS) and the Oswestry Disability Index (ODI). Only patients who achieved a positive response during the trial stimulation period were included in the final analysis.

A follow-up assessment was performed between 3 and 6 months after completion of the surgical treatment. Patients completed the questionnaires independently, without the presence of the operating surgeon, to minimize potential bias related to physician influence on patient-reported outcomes.

### 2.4. Statistical Procedures

Categorical variables were presented as integer numbers and percentages (frequencies). Numerical variables are expressed as means, medians, standard deviations, and lower-to-upper quartile values. The normality of distribution was assessed using the Shapiro–Wilk W test. Due to the small sample size, non-parametric procedures were performed for repeated measurements using the Wilcoxon signed-rank test. A level of *p* < 0.05 was considered statistically significant. Statistical tests were selected based on data distribution and the paired nature of pre- and post-treatment comparisons. Given the exploratory nature of the study and the limited sample size, no adjustment for multiple comparisons was performed. No multivariable analysis was conducted due to the relatively small sample size; therefore, the results should be interpreted as descriptive and hypothesis-generating. Continuous variables were compared using paired tests (paired *t*-test or Wilcoxon signed-rank test, depending on data distribution), while categorical variables were analyzed descriptively. No a priori sample size calculation was performed, as this was an exploratory study based on a consecutive series of patients treated during the study period. All the analyses were performed using Statistica™, release 13.3 (TIBCO Software Inc., Palo Alto, CA, USA).

## 3. Results

The study population included patients with chronic spinal pain of predominantly neuropathic origin, including both postsurgical spine syndrome and degenerative spinal disease. Imaging studies (MRI/CT) were performed in all patients to exclude surgically treatable pathology. During the study period, a total of 42 patients underwent SCS implantation in the Department of Neurosurgery. One patient was excluded from analysis due to damage to the percutaneous electrode and the need for delayed replacement with a surgically implanted electrode, resulting in a final cohort of 41 patients. Only patients who had undergone successful trial stimulation with a percutaneously implanted electrode were included in the analysis.

Women predominated in the study group, constituting 88% of the cohort (37 out of 42 patients), whereas only five men underwent surgery during the study period. This predominance of female patients reflects the epidemiology of chronic pain syndromes, which are more prevalent and often more severe in women. Sex-related differences in pain perception, central sensitization, and responsiveness to neuromodulation have been reported previously and may partially explain the observed gender distribution.

The age of patients ranged from 50 to 92 years, with more than 50% of patients undergoing surgery in the 60–75-year age group (mean age: 68.93 years) (Table 1). The wide age range reflects the chronic and progressive nature of pain syndromes eligible for spinal cord stimulation, as well as the absence of a strict upper age limit for neuromodulation when appropriate patient selection is applied. Chronological age alone was not considered a contraindication for SCS implantation. Inclusion of elderly patients increases the external validity of the study and supports the feasibility of SCS therapy in an aging population.

The final analysis included 41 patients over the age of 50 who were not eligible for conventional spinal surgery due to the absence of clear surgical indications, such as herniated nucleus pulposus, spinal stenosis, or spinal instability (52.5% of patients), had previously undergone unsuccessful surgical treatment (15.8%, consistent with persistent spinal pain syndrome), or could not undergo surgery due to its invasiveness and the presence of significant medical comorbidities (31.7%).

**Figure 1:**
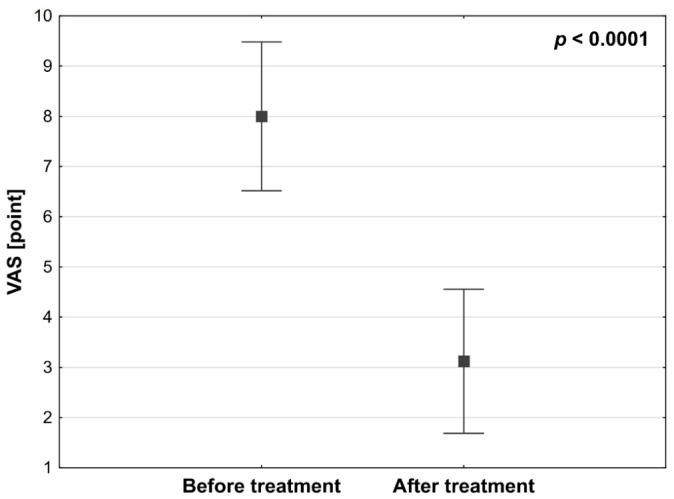
Pain relief according to the VAS in the study cohort before and after using stimulators (n = 41) (*p* < 0.0001).

**Figure 2:**
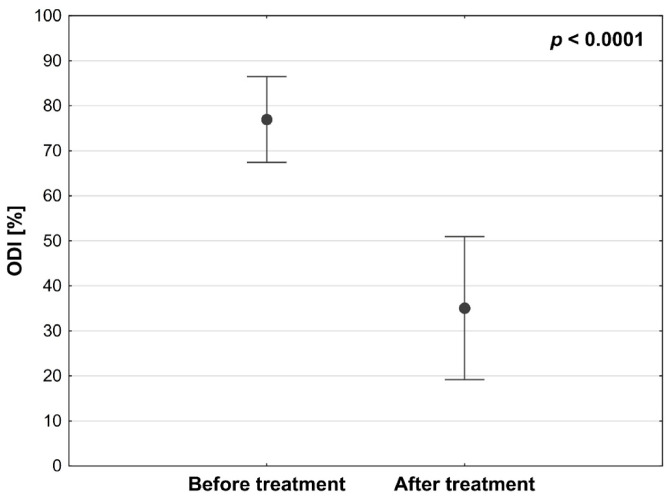
Disability amelioration according to the ODI in the study cohort before and after using stimulators (n = 41) (*p* < 0.0001).

In the study group, a statistically significant reduction in perceived pain was observed after surgical treatment (*p* < 0.0001). Median pain intensity measured using the Visual Analog Scale decreased from 8 before treatment to 3 after SCS implantation (Figure 1). Similarly, functional disability improved significantly, with the median Oswestry Disability Index decreasing from 74% preoperatively to 38% following SCS implantation (Figure 2).

Age-stratified analysis demonstrated that the greatest reduction in pain intensity (VAS) was observed in the oldest subgroup of patients aged over 75 years, which constituted 31.7% of the cohort. In contrast, the most pronounced improvement in functional status, as measured by the Oswestry Disability Index, was observed in the 65–75-year age group (46.3% of the cohort) (Table 2).

Effect size measures: VAS, Z = 5.579, r = 0.86, *p* < 0.0001; ODI, Z = 5.511, r = 0.87, *p* < 0.0001.

Statistical power: VAS, >0.9999; ODI, >0.9999.

Age grouping:

- 50–64 years, nine subjects (21.95%);
- 65–74 years, nineteen subjects (46.34%);
- 75+ years, thirteen subjects (31.71%).

Effect size measures:

VAS:

- 50–64 years, Z = 2.666, r = 0.89, *p* = 0.0077;
- 65–74 years, Z = 3.823, r = 0.88, *p* = 0.0001;
- 75+ years, Z = 3.180, r = 0.88, *p* = 0.0015.

ODI:

- 50–64 years, Z = 2.666, r = 0.89, *p* = 0.0077;
- 65–74 years, Z = 3.724, r = 0.85, *p* = 0.0002;
- 75+ years, Z = 3.180, r = 0.86, *p* = 0.0015.

Statistical power:

VAS:

- 0.9984;
- >0.9999;
- >0.9999.

ODI:

- 0.9922;
- >0.9999;
- 0.9996.

A reduction in the use of analgesics and anti-inflammatory medications was observed in all patients following SCS implantation. Notably, patients who had required opioid analgesics prior to treatment were able to discontinue their use after the procedure. Due to the retrospective nature of medication reporting, detailed quantitative data on analgesic use were not consistently available, which represents a limitation.

**Figure 3:**
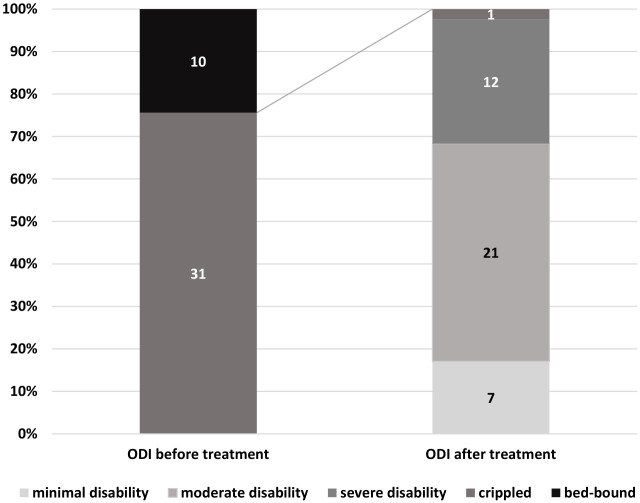
Disability classification according to the ODI in the study cohort before and after using stimulators (n = 41) (*p* < 0.0001).

Functional status, assessed using the Oswestry Disability Index (ODI) categories, improved substantially after treatment. At baseline, 31 patients were classified as crippled (75.61%), while 10 patients were bed-bound (24.39%). Following SCS implantation, functional status improved markedly: 7 patients were classified as minimally disabled (17.07%), 21 as moderately disabled (51.22%), and 12 as severely disabled (29.27%). Only one patient remained in the crippled category (2.44%) (Figure 3).

These findings indicate a substantial shift from the most severe disability categories toward moderate and minimal disability following neuromodulation therapy.

Effect size measures: Chi^2^ = 78.125, df = 4, *p* < 0.0001.

Observed statistical power: 0.9999.

The observed reduction in pain intensity exceeded the minimal clinically important difference (MCID) for the VAS, which is generally considered to be a reduction of at least 2 points or 30%. In our cohort, the median decrease in VAS score was 5 points, corresponding to a 62.5% reduction in pain intensity.

Similarly, the improvement in functional disability significantly exceeded the MCID for the Oswestry Disability Index, typically estimated at 10–12 points. The median ODI improvement in our study was 36 points, indicating a substantial clinical benefit.

It should be noted that the enrichment design of the study, including only patients with a positive response to trial stimulation, may contribute to a higher proportion of patients exceeding the MCID threshold.

Adverse events were monitored during the perioperative period and follow-up visits based on clinical assessment. No procedure-related adverse events were observed. One device-related complication occurred, consisting of damage to the percutaneous electrode. This patient was excluded from the final analysis and was subsequently scheduled for implantation of a surgically placed electrode.

A patient flow diagram summarizing the study cohort and selection process is provided in Supplementary Figure S1.

## 4. Discussion

Spinal cord stimulation (SCS) is considered an effective treatment modality for chronic lumbar spine pain and has demonstrated particular efficacy in the management of neuropathic pain and persistent spinal pain syndrome (PSPS) [11,12]. Several mechanisms underlying the analgesic effects of SCS have been proposed, primarily involving modulation and inhibition of nociceptive signal transmission within the dorsal columns of the spinal cord.

The development of SCS was initially based on the gate control theory of pain, which partially explains the analgesic effect observed with conventional tonic stimulation [13]. However, the precise mechanisms responsible for the therapeutic effects of SCS remain incompletely understood, and additional pathways are currently being investigated. This study focused on patients with predominantly neuropathic pain, which is known to respond more favorably to neuromodulation therapies such as SCS.

Recent studies suggest that patients with chronic pain may exhibit elevated concentrations of pro-inflammatory mediators in the cerebrospinal fluid. Interestingly, individuals treated with spinal cord stimulation have been shown to demonstrate lower levels of several inflammatory cytokines, including interleukin-6 (IL-6), interleukin-1 (IL-1), vascular endothelial growth factor (VEGF), and tumor necrosis factor-alpha (TNF-α) [14]. Moreover, the balance between pro- and anti-inflammatory mediators within the spinal cord may play an important role in the modulation of chronic pain [15]. These findings suggest that neuroinflammatory modulation may represent an additional mechanism contributing to the effectiveness of newer stimulation paradigms, such as burst stimulation and high-frequency stimulation (HF-SCS), compared with conventional tonic stimulation.

The introduction of burst stimulation represents an important advancement in neuromodulation therapy. Unlike tonic stimulation, burst stimulation does not require patients to perceive paresthesia, which some individuals describe as uncomfortable or unpleasant [16]. Furthermore, several studies have demonstrated superior clinical outcomes with burst stimulation compared with conventional tonic stimulation. For example, the SUNBURST study, conducted in a cohort of 100 patients, demonstrated improved pain relief with burst stimulation relative to traditional tonic SCS [17]. Another potential advantage of burst stimulation is improved energy efficiency, which may translate into longer battery life of the implanted pulse generator and reduce the need for surgical replacement procedures.

The magnitude of pain reduction and functional improvement observed in our cohort appears consistent with outcomes reported in randomized trials such as the DISTINCT study and with prior evidence supporting burst stimulation, including the SUNBURST trial. However, direct comparisons remain limited due to differences in study design, patient selection, enrichment strategies following trial stimulation, and follow-up duration. Unlike randomized controlled trials, our study reflects real-world clinical practice in a selected population of non-surgical candidates.

Deer et al. compared the effectiveness of spinal cord stimulation with conservative treatment in patients with chronic low back pain [18]. Their results demonstrated a significant therapeutic benefit of SCS, with more than a 70% reduction in pain intensity measured using the Numeric Rating Scale (NRS). In contrast, patients receiving conventional conservative treatment achieved only a modest reduction in pain (slightly above 7% on the NRS). Furthermore, 91% of patients in the SCS group reported clinically meaningful pain improvement compared with only 16% in the control group [18].

In our study, similarly high and statistically significant effectiveness of SCS therapy was observed. Treatment with burst SCS resulted in a reduction in pain intensity measured using the Visual Analog Scale (VAS), from a mean value of 8 before treatment to 3.12 after implantation. Additionally, functional status improved substantially, with the mean Oswestry Disability Index (ODI) decreasing from 76.95% to 35.12%. Importantly, the magnitude of pain reduction clearly exceeded commonly accepted thresholds for minimal clinically important difference (MCID) in chronic pain treatment, which are typically defined as a reduction of approximately 2 points on the VAS or a relative reduction of at least 30%. These findings indicate that SCS implantation not only produced statistically significant results but also achieved clinically meaningful improvement in patient outcomes.

James J. Yue et al. evaluated the effectiveness of spinal cord stimulation in patients without structural pathology visible on MRI that would qualify them for surgical intervention. In our study, this subgroup constituted 38% of the analyzed patient population [19]. In the study by Yue et al., a clinical response—defined as a reduction in pain intensity of more than 50% compared with baseline—was achieved in 85.3% of patients [19]. In the control group, more than 66% of participants opted to undergo SCS implantation after six months, ultimately achieving a positive response rate of 71.4% during the 12-month follow-up period [19].

Similarly, a meta-analysis conducted by Mi Zhou et al. compared spinal cord stimulation with conservative treatment and demonstrated significant effectiveness of SCS in reducing pain intensity measured using the VAS and improving outcomes assessed with the McGill Pain Questionnaire [20].

However, some studies comparing SCS with placebo have questioned the magnitude of its therapeutic effect. Perruchoud et al., in a double-blind study, suggested that during the early postoperative period, pain improvement may occur at a similar level in patients with the stimulator turned on and off. The authors also reported comparable pain reduction in the high-frequency stimulation group and the control group during longer-term follow-up. Nevertheless, the study was conducted in a relatively small patient cohort. Although a trend toward improved outcomes in the HF-SCS group was observed, the difference did not reach statistical significance [21].

The present prospective study demonstrates a statistically significant reduction in pain intensity and disability following SCS implantation in a cohort of patients with chronic neuropathic spinal pain. The results should be interpreted as exploratory and hypothesis-generating, particularly in the context of the enriched study design. In nearly all treated patients, a reduction in the use of analgesic medications was achieved following therapy. Notably, all patients who had previously required opioid analgesics were able to discontinue their use after SCS implantation.

The present prospective study demonstrated both statistically and clinically significant reductions in pain intensity and disability following spinal cord stimulation in patients with chronic neuropathic spinal pain. The observed decrease in median VAS scores from 8 to 3 and the reduction in Oswestry Disability Index from 74% to 38%, accompanied by decreased analgesic consumption, indicate a meaningful therapeutic effect in this difficult-to-treat population.

Several methodological strengths enhance the internal validity of this study. The prospective design allowed for predefined inclusion criteria and standardized follow-up. The findings should be interpreted in the context of an enriched cohort of trial responders. A uniform two-stage percutaneous implantation technique and consistent use of burst stimulation minimized procedural variability. Furthermore, validated outcome measures were applied systematically at baseline and follow-up, and treatment effectiveness was evaluated not only through pain reduction but also through functional improvement and decreased pharmacological burden.

Nevertheless, several limitations should be acknowledged. This was a single-center study with a relatively small sample size, which may limit statistical power and generalizability. The absence of a randomized control group precludes definitive conclusions regarding comparative effectiveness and does not allow full exclusion of placebo effects, regression to the mean, or natural variability in pain perception. Additionally, the enrichment design introduces selection bias and may overestimate treatment effectiveness. The relatively short follow-up period (3–6 months) limits the assessment of long-term outcomes. Finally, reliance on patient-reported outcome measures may introduce subjective bias, and detailed quantitative data on analgesic dosing were not consistently available. Despite these limitations, the prospective design and consistent treatment protocol strengthen the internal validity of the findings.

Taken together, our findings support spinal cord stimulation as an effective and minimally invasive therapeutic option for carefully selected patients with chronic neuropathic spinal pain, particularly those who are not suitable candidates for conventional spinal surgery. However, larger randomized controlled trials with longer follow-up are required to further define long-term efficacy, cost-effectiveness, and optimal patient selection criteria.

## 5. Conclusions

Burst spinal cord stimulation was associated with significant short-term clinical benefit in this single-center cohort of patients with chronic neuropathic spinal pain. The minimally invasive percutaneous implantation technique allows the procedure to be performed without the need for extensive surgery or general anesthesia, making it a valuable therapeutic option for patients who are not candidates for conventional spinal procedures. SCS was also highly effective in patients with persistent spinal pain syndrome and enabled discontinuation of opioid analgesics in this group. Nevertheless, longer follow-up and larger prospective studies are needed to confirm the durability of these outcomes and further refine patient selection. These findings suggest that burst spinal cord stimulation may be an effective treatment option in carefully selected patients; however, further studies with larger cohorts and longer follow-up are required to confirm these results.

## Figures and Tables

**Table 1:** Characteristics of the study cohort before and after using stimulators (n = 41).

Analyzed Trait	Treatment Phase	Statistical Parameter *					*p*-Value
		M	SD	Me	Q_1_–Q_3_	95% CI	
Age [y]		68.93	8.94	70	65–75	66.10–71.75	
VAS [point]	Before	8.00	1.48	8	7–9	7.53–8.47	<0.0001
	After	3.12	1.43	3	2–4	2.67–3.57	
	Abs. Δ	4.88	2.23	5	4–6	4.17–5.58	
ODI [%]	Before	76.95	9.53	74	70–80	79.94–79.96	<0.0001
	After	35.07	15.89	38	24–46	30.06–40.09	
	Abs. Δ	41.88	21.55	40	28–54	35.07–48.68	

* Statistical measures used: M—mean; SD—standard deviation; Me—median; Q—quartiles; CI—confidence interval; VAS—Visual Analog Scale; ODI—Oswestry Disability Index; Abs.—absolute number.

**Table 2:** Changes in the investigated traits due to treatment in the study by age group (n = 41).

Analyzed Trait	Age Group	Treatment Phase	Statistical Parameter *					*p*-Value
			M	SD	Me	Q_1_–Q_3_	95% CI	
VAS [point]	50–64	Before	8.00	1.58	8	7–9	6.78–9.22	0.0077
		After	3.67	1.68	4	4–4	2.39–4.94	
		Abs. Δ	4.33	2.29	4	2–5	2.57–6.09	
	65–74	Before	8.26	1.28	8	7–9	7.64–8.88	0.0001
		After	3.10	1.37	3	2–4	2.44–3.77	
		Abs. Δ	5.16	2.09	5	4–6	4.15–6.16	
	75+	Before	7.62	1.71	8	6–9	6.58–8.65	0.0015
		After	2.77	1.36	3	2–4	1.94–3.59	
		Abs. Δ	4.85	2.48	4	3–7	3.35–6.34	
ODI [%]	50–64	Before	79.11	12.33	80	70–90	69.63–88.59	0.0077
		After	40.00	17.66	40	32–64	26.42–53.58	
		Abs. Δ	39.11	23.26	34	24–40	21.23–56.99	
	65–74	Before	77.00	7.98	78	74–84	73.15–80.85	0.0002
		After	31.58	15.64	30	22–44	24.03–39.12	
		Abs. Δ	45.42	19.62	44	30–58	35.96–54.88	
	75+	Before	75.38	9.98	72	70–78	69.35–81.42	0.0015
		After	36.77	15.04	40	30–50	27.68–45.85	
		Abs. Δ	38.61	23.94	30	24–42	24.15–53.08	

* Statistical measures used: M—mean; SD—standard deviation; Me—median; CI—confidence interval; Q—quartiles; VAS—Visual Analog Scale; ODI—Oswestry Disability Index; Abs.—absolute number.

## Data Availability

The datasets generated and analyzed during the current study are not publicly available due to patient privacy and ethical restrictions, but they are available from the corresponding author upon reasonable request.

## Supplementary Materials

The following supporting information can be downloaded at: https://www.mdpi.com/article/10.3390/medicina62050816/s1, Figure S1: Patient flow diagram illustrating the study population, trial stimulation phase, exclusions, and final cohort included in the analysis.
