# Supplementary figures and images for: Effectiveness of Spinal Cord Stimulation in the Treatment of Lumbar Spine Pain Syndromes
Source: Medicina (Kaunas). 2026 Apr 24;62(5):816. doi: 10.3390/medicina62050816 (PMC13208511; doi:10.3390/medicina62050816)

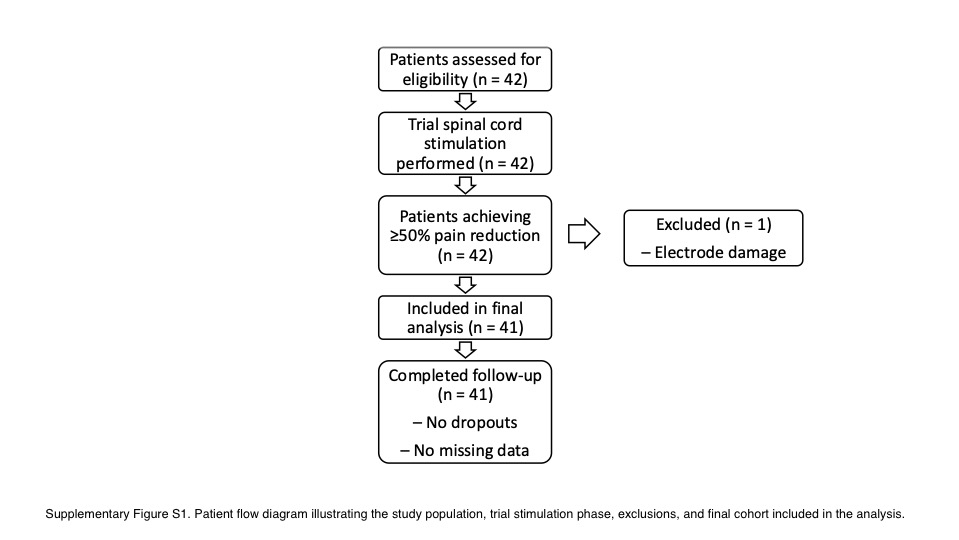

Supplement: Supplementary file 1 [file medicina-62-00816-s001.zip › medicina-4220083-supplementary.jpg]
